# A longitudinal study of bovine viral diarrhea virus in a semi-closed management dairy cattle herd, 2020–2022

**DOI:** 10.3389/fvets.2023.1221883

**Published:** 2023-09-14

**Authors:** Abdullah I. A. Al-Mubarak, Anwar A. G. Al-Kubati, Abdullah Skeikh, Jamal Hussen, Mahmoud Kandeel, Baraa Flemban, Maged Gomaa Hemida

**Affiliations:** ^1^Department of Microbiology, College of Veterinary Medicine, King Faisal University, Al-Hofuf, Saudi Arabia; ^2^Department of Veterinary Medicine, Faculty of Agriculture and Veterinary Medicine, Thamar University, Dhamar, Yemen; ^3^Camel Research Center, King Faisal University, Al-Ahsa, Saudi Arabia; ^4^Department of Biomedical Sciences, College of Veterinary Medicine, King Faisal University, Al-Hofuf, Saudi Arabia; ^5^Department of Pharmacology, Faculty of Veterinary Medicine, Kafrelsheikh University, Kafrelsheikh, Egypt; ^6^Department of Veterinary Biomedical Sciences, College of Veterinary Medicine, Long Island University, Brookville, NY, United States

**Keywords:** bovine viral diarrhea virus (BVDV), surveillance, ELISA, RT-PCR, longitudinal study, persistent infection, transient infection

## Abstract

**Introduction:**

Bovine viral diarrhea virus (BVDV) brings great economic loss to the cattle industry worldwide. Developing a control/prevention strategy requires the prior assessment of certain epidemiological parameters. To determine the BVD incidence rate and associated risk factors, a dairy cattle herd in the eastern region of Saudi Arabia was monitored between 2020 and 2022.

**Methods:**

Nasal swabs (*n* = 190), rectal swabs (*n* = 190), and sera (*n* = 190) were collected from 79 cows in this herd. Collected sera and swabs were tested using the commercially available ELISAs for the BVDV antibodies and antigens, respectively. Collected sera were also tested for the presence of BVDV nucleic acids using commercial real-time RT-PCR kits.

**Results and discussion:**

Our data show BVDV seroprevalence (18.8%, 15%, and 8.2%) in the tested animals in 2020–2022, respectively. None of the collected nasal swabs, rectal swabs, or sera tested positive for the BVDV antigen, whereas 10.1%, 10%, and 18.1% of the tested sera were positive for BVDV nucleic acid in 2020–2022, respectively. The incidence rate was estimated at 0.02446 new cases/year despite the detection of BVDV in seronegative animals on single or two occasions at ≥6-month intervals. Young calves and bulls remained apparently unexposed to BVDV despite their presence with BVDV-infected females, with no significant physical separation. Both seropositivity and nucleic acid detectability showed significant positive and negative correlations, respectively, with reproductive performance. Collectively, the present study provides useful clues about the transmissibility of BVDV in the presence of possibly persistently infected animals. To the best of our knowledge, this is the first longitudinal study of BVDV in the Eastern Region of Saudi Arabia. Further detailed characterization of the circulating BVDVs is encouraged.

## 1. Introduction

Bovine viral diarrhea (BVD) is a widely distributed viral infection among various domestic and wild species of animals. Cattle are the most significantly affected species. Infection with the BVD virus (BVDV) in cattle induces various clinical syndromes such as respiratory, reproductive, digestive, and/or immunosuppressive manifestations (1). It causes considerable economic losses to the cattle industry worldwide. Direct losses have been estimated at 42.14€ per animal per year (2). These losses include mortality, morbidity, a decrease in productivity, and reproductive problems such as abortion, stillbirth, congenital malformation, and reproductive failure. Additionally, indirect loss occurs due to the cost of control/prevention measures (2). BVDV belongs to the genus *Pestivirus* of the family *Flaviviridae*. Three species have been identified, including Pestivirus A (BVDV-1), Pestivirus B (BVDV-2), and Pestivirus H (Ho-Bi virus, or BVDV3). The virus also possesses a close antigenic relationship to the classical swine fever virus (Pestivirus C) and the border disease virus (Pestivirus D) (3). BVDV has a single-stranded, positive-sense RNA genome of ~12.3 kb in length. It is composed of a single open reading frame (ORF) flanked by two untranslated terminal regions (UTRs), namely the 5'UTR and the 3'UTR. Translation of the BVDV genome results in a single polyprotein that is subsequently cleaved into four structural proteins (C, Erns, E1, and E2) and eight non-structural proteins (N^pro^, p7, NS2, NS3, NS4A, NS4B, NS5A, and NS5B) (3). Regions encoding the E2, N^pro^, and 5'UTR have been frequently used for genotyping the BVDV. On the other hand, the bio-typing of BVDV depends mainly on the induced cytopathic effects of the virus in cell culture. Based on this cytopathology induction criterion, BVDV is categorized into two types: cytopathic (CP) and non-cytopathic (NCP) (4).

BVDV biotypes behave differently in the virus–host interaction. The NCP-BVDV usually induces a transient infection (TI) that is mild compared to that induced by the CP-BVDV strains (5). BVDV infection in pregnant cattle with the NCP strains during the period between 42 and 114 days of gestation may result in the delivery of persistently infected (PI) calves. These calves are seronegative and immunotolerant to the subsequent infection with homologous BVDV strains (6). The BVDV-PI animals usually shed the virus during their entire lives and represent the main source of infection with the NCP-BVDV. This is in contrast to animals with TI that shed the BVDV for a relatively short period of ~14 days (6–10). Consequently, detection and elimination of the BVDV-PI animal are the key factor for the control/eradication of the BVDV from cattle populations. Practically, the BVDV-PI animal is an animal that tested positive for BVDV in two consecutive samples that were collected ≥3 weeks apart (11, 12). Detection of BVDV may be conducted using several laboratory methods (13); however, antigen capture (AC)-ELISA and RT-PCR are the commonly used techniques for the detection of BVDV antigen and nucleic acids, respectively (14). On the other hand, competitive and indirect ELISA tests are commonly used for the detection of anti-BVDV antibodies. These assays are commercially available and have been evaluated for their performance on various types of samples, including skin, buffy coat, serum, milk, and various types of cattle swabs (nasal, oral, conjunctival, or vaginal) (14–18).

The prevalence of BVDV in cattle populations may vary according to several factors, including, but not limited to, the production type, management, population density, age and health status of the sampled animals, the implemented control/eradication programs, the vaccination coverage, and the sensitivity/specificity of the used BVDV diagnostic assays (10, 19). Globally, a meta-analysis of the BVDV prevalence studies showed that, at the animal level, the prevalence of BVDV-PI animals varied from low (≤ 0.8%) in Europe and Australia to high (>1.6%) in West Asia. The prevalence was higher in the countries that failed to apply rigorous control/eradication strategies against BVDV infections (10). In the Middle East, the prevalence of BVDV-PI animals was 1.5% and 0.8% in Egypt and Iraq, respectively. Based on AC-ELISA, the prevalence of BVDV was approximately 6% in Egypt and Iraq, while it was approximately 0.3% in the United Arab Emirates (20–23). Molecular BVDV surveillance based on RT-PCR revealed that ~7.5% and 10% to 14% of the tested animal populations in Egypt and Iraq, respectively, were BVDV-positive (20–22). On the other hand, the seroprevalence of BVDV is heavily affected by the vaccination coverage of cattle populations. The seroprevalence of BVDV in Europe was reported to vary between 60% and 85% (10). In the Middle East, seroprevalence varies from 40% in Egypt (24) to approximately 25% in Sudan (25), Iraq (22), and Iran (26). Similarly, a seroprevalence of 26% in Saudi Arabia and 35% in both Saudi Arabia and Oman was previously reported (27–30). Our previous serosurveillance of BVDV on non-bovine species of animals in the Eastern Region of Saudi Arabia showed seroprevalence of 4.5% in camels and 3.5% in goats, whereas none of the tested sheep sera were positive for anti-BVDV antibody, while AC-ELISA showed none of the tested samples was BVDV-positive (31).

## 2. Materials and methods

### 2.1. Animal ethics approval

The study was approved by the Animal Ethics Committee of King Faisal University (approval no. KFU-REC/2020-12-36).

### 2.2. Cattle herd description

The longitudinal study was conducted on a dairy cattle herd kept under a semi-closed management system in the Eastern Region of Saudi Arabia during the period from 2020 to 2022. All the necessary paperwork for sample collection was approved. The cattle herd was kept in a wire-fenced, gated facility and divided into three sections separated by string-wire fences (newborn animals, males, and females). Animals in these sections shared the source of food and water, and the same staff, including veterinarians and employees, were dealing with the three compartments at the same time in a daily routine practice. The herd was in proximity to other camel herds as well as sheep and goat farms with wire fence separation. At the beginning of the current study, this herd consisted of 69 animals, including 16 calves (< 1 year old) and 53 adult animals. There was incidental removal and introduction of some animals during the tenure of this study, and collectively, 79 animals were included in the study. During the study, 15 animals (3 calves and 12 adults) died, mostly with signs of diarrhea and septicemia (*n* = 10).

### 2.3. Sample collection and processing

Blood samples were collected by venipuncture of the jugular vein of the involved animals into plan vacuum tubes. The collected samples were kept at 4°C overnight. Samples were then centrifuged (Eppendorf, Hamburg, Germany) at 1,000 × g for 15 min, and sera were transferred into sterile screw-capped 2 mL tubes and stored at (−20°C) until further testing. The nasal swabs were collected by introducing sterile cotton swabs deep into the nasal cavity to touch the back of the nasal septa. After being soaked in mucosal secretion, swabs were transferred into clean, sterile tubes containing viral transport media (Sigma, USA). The rectal swabs were collected by introducing the cotton swabs into the rectum to collect some of the rectal secretions and fecal materials. The downstream procedure for processing both types of swabs is similar. Both types of swabs were transported on ice into our laboratory. Thereafter, each swab was vigorously vortexed and then centrifuged at 5,000 × g for 5 min at 4°C. The clear supernatants were collected in sterile 2 mL screw-capped tubes and stored at −80°C until further testing.

### 2.4. Detection of anti-BVDV antibodies in the collected cattle sera

The commercial ID Screen^®^ BVD p80 Antibody Competition ELISA kit (Cat. # BVDC-5P, ID-Vet, France) was used for the detection of anti-BVDV antibodies in the collected cattle sera. A final dilution of 1:100 was prepared from each serum sample in PBS dilution buffer, and 100 μl of the diluted sera, negative control serum, or positive control serum were added to the wells of microtiter plates pre-coated with the BVD viral protein P80-125. After incubation for 45 min at 37°C, the plates were washed three times with 300 μl/well of the washing buffer. A 100 μl volume of peroxidase-conjugated anti-BVDV P80-125 antibody was added to each well, followed by incubation for 30 min at 21°C. After three washes, the substrate-chromogen solution was added to the plates, and the plates were incubated for 30 min at 21°C in the dark. Finally, the stop solution was added to the plates, and the color density was measured using a spectrophotometer (Bio-Rad, Watford, United Kingdom) at 450 nm. The test was considered valid if the optical density value of the negative control (ODnc) was >0.7, and the mean value of the positive control (ODpc) was < 30% of the ODnc. For each sample, the competition percentage (S/N%) was calculated by dividing the optical density value of the sample by the ODnc and multiplying the result by 100 (OD-sample/ODnc x 100). A competition percentage equal to or < 40% was considered positive. A competitive percentage equal to or more than 50% was considered negative. A competition percentage between 40% and 50% was considered doubtful.

### 2.5. Detection of BVDV antigen in collected serum and swab samples

The BVDV protein 80 (P80) antigen ELISA kit (Cat. # BVDAGP80-5P, ID-Vet) was used for the detection of the BVDV antigen in collected serum and nasal swab samples. A final dilution of 1:2 was prepared from each serum sample in PBS dilution buffer. A volume of 100 μl of the diluted serum samples, undiluted elutes of the nasal swabs, negative control serum, and positive control serum were added to the wells of microtiter plates pre-coated with capture antibodies against the BVDV P80-125. After incubation for 60 min at 37°C, the plates were washed five times in the washing buffer. Thereafter, a volume of 100 μl of the peroxidase-conjugated anti-BVDV P80-125 detection antibody was added to each well, followed by incubation for 30 min at 37°C. After three washes, the substrate-chromogen solution was added to the plates, and the plates were incubated for 30 min at 21°C in the dark. Finally, the stop solution was added to the plates, and the color density was measured using a spectrophotometer (Bio-Rad) at 450 nm. The test was considered valid if the optical density value of the positive control was higher than 0.500, and the ratio of the mean values of the positive and negative controls (ODpc/ODnc) was >3. For each tested sample, the percentage of positive control (S/P%) was calculated. An S/P% of < 35% was considered negative. An S/P% equal to or >35% was considered positive.

### 2.6. RNA extraction

A total of 207 samples were tested for the presence of BVDV nucleic acids, including 162 samples collected during 2020 and 2021 (129 sera and 33 swabs) and 45 samples collected in 2022 (27 sera and 18 swabs). Samples were divided into batches, with each batch having 4–9 samples. Samples from positive batches were retested individually.

We used TRIzol to extract the RNA from serum and swab samples. Each tube of serum was pelleted with high-speed centrifugation (14,000 x g for 15 min). The supernatant was discarded, and 1 ml of the TRIzol reagent was added to the pellet. Tubes were then vortexed for 15 s and incubated for 5 min at room temperature. After that, 0.2 ml of chloroform solution was added to the mixture, followed by vortexing for 15 s and incubation for 5 min at room temperature. This lysate was centrifuged at 12,000 x g for 15 min at 4°C. The aqueous phase was collected in new tubes without disturbing the interphase and mixed with 0.5 ml of isopropanol. The mixture was mixed by inversion and then incubated for 10 min at room temperature. All the tubes were centrifuged at 12,000 x g for 10 min to precipitate the RNAs, washed with 75% ethanol twice, and allowed to air dry. RNAs were then dissolved in DEPC-treated water, quantified using a NanoDrop 2000c spectrophotometer (Thermo Fisher Scientific Inc.), and stored at (−20°C).

### 2.7. Detection of the BVDV nucleic acids by the real-time RT-PCR technique

The real-time RT-PCR kit, ID gene BVD/BD triplex (Cat # IDBVDV2-100, ID-vet), was used to detect BVDV nucleic acid in collected serum and nasal swab samples. Provided controls, including target positive control (TPC), target positive control-ear notch sample (TPC-EN), non-target positive control (NTPC), and negative extraction control (NEC), were subjected to the RNA extraction step along with samples in order to assess the efficiency of the extraction and the presence of PCR inhibitors. The real-time PCR machine VII7A Life Technologies, Applied Biosystems, was employed along with FAM (525 nm), VIC, or Yakima yellow (548 nm), and Cy5 (650 nm) dyes. Amplification was performed with a mixture containing 8 μL of the master mix and 5 μL of RNA extract or nuclease-free water in the case of a negative control for amplification (NAC). The real-time RT-PCR program was set for 42 cycles: the first for reverse transcription (10 min at 45°C), the second for polymerase activation (10 min at 95°C), and 40 cycles of RNA denaturation (15 s at 95°C), and finally, elongation (60 s at 60°C).

### 2.8. Data analysis

The incidence rate, defined as the number of susceptible animals that became diseased per unit of time, was calculated using the following formula (32): Incidence rate = (Number of new cases in the study period)/(total time for animals at risk). The calculation involved animals that initially tested seronegative and had one or more succeeding tests. For seroconverted animals, half of the days of the season during which they were seroconverted were included in the denominator (32). To assess the significance of the association between BVDV seropositivity/detectability and some risk-associated factors, a chi-square test was performed and presented in the results section. As the chi-square test is not recommended for small values, the recommended alternative, Fisher's exact test, was used with the Freeman–Halton extension (2 X 3 or 2 X 4 contingency tables). The two-tailed *P*-values are shown unless stated otherwise. The more powerful and less conservative “mid *P*-value” recommended by Armitage and Berry was also considered (33).

## 3. Results

### 3.1. Seroprevalence of BVDV in the studied cattle herd, 2020–2022

**Figure 1:**
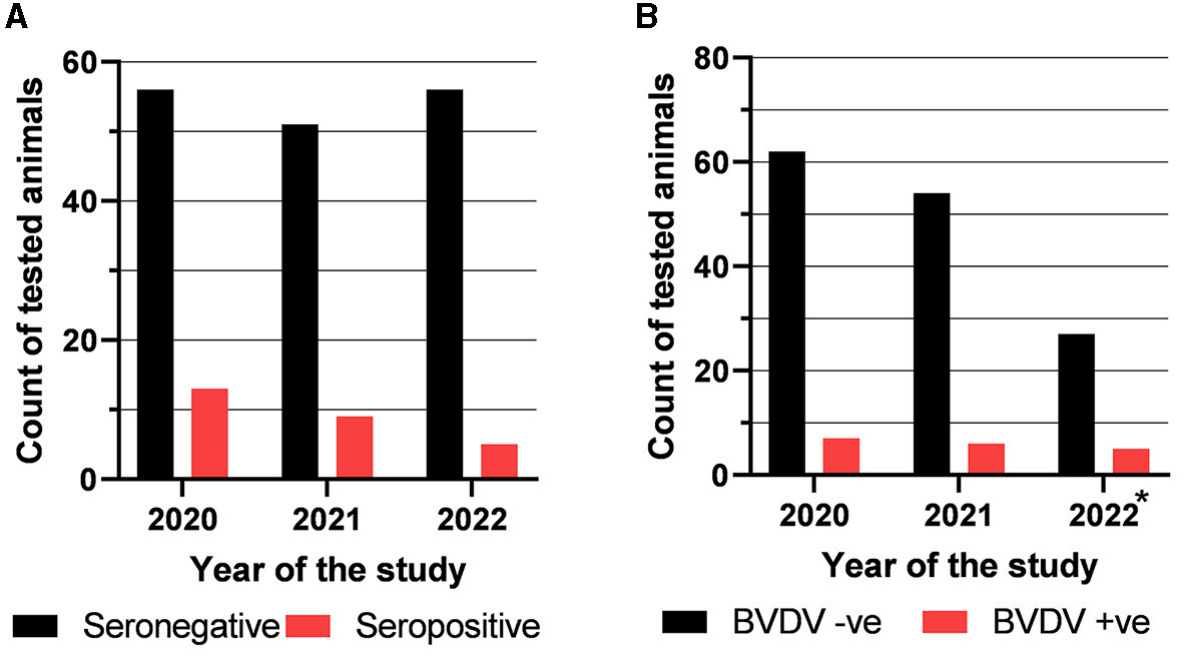
Results of testing of the collected samples with competitive ELISA to detect anti-BVDV antibody **(A)** and with real-time RT-PCR to detect BVDV nucleic acid **(B)**. *Samples of the batches that tested positive during the 2020 or 2021 seasons continued to be tested in the last season (2022).

The obtained serology results showed a decreasing trend in seropositivity. Out of the 69 collected and tested samples in 2020, there were 13 (18.84%) that were seropositive. Nine (15%) out of the 60 samples collected during 2021 were seropositive, while only 5 out of the 61 samples collected during 2022 were seropositive for BVDV (8.2%) (Figure 1A).

### 3.2. Molecular detection of BVDV in collected sera and swabs by real-time RT-PCR

The collected cattle sera were also tested for the presence of BVDV nucleic acid using real-time RT-PCR. The results revealed that 7 out of 69 (10.15%) sampled cows in the year 2020 were BVDV-positive. In the year 2021, 6 out of 60 sampled cows were BVDV-positive (10%), as shown in Figure 1B. Samples of the batches that tested positive during the 2020 or 2021 seasons continued to be tested in the last season (2022). Five out of 27 tested samples (18.52%) were BVDV-RNA positive. Notably, none of the tested nasal or rectal swabs were BVDV-positive.

### 3.3. Serological detection of BVDV in collected cattle sera and nasal swabs

The analysis of serum samples and nasal swabs (129 samples of each type) collected in 2020 and 2021 from adults (*n* = 99) and newborns (*n* = 30) using a sandwich-ELISA test for the BVDV viral protein 80 (P80) revealed no detectable levels of the viral antigen in any of the serum or swab samples.

### 3.4. Findings of the longitudinal study of BVDV in the targeted cattle herd

**Figure 2:**
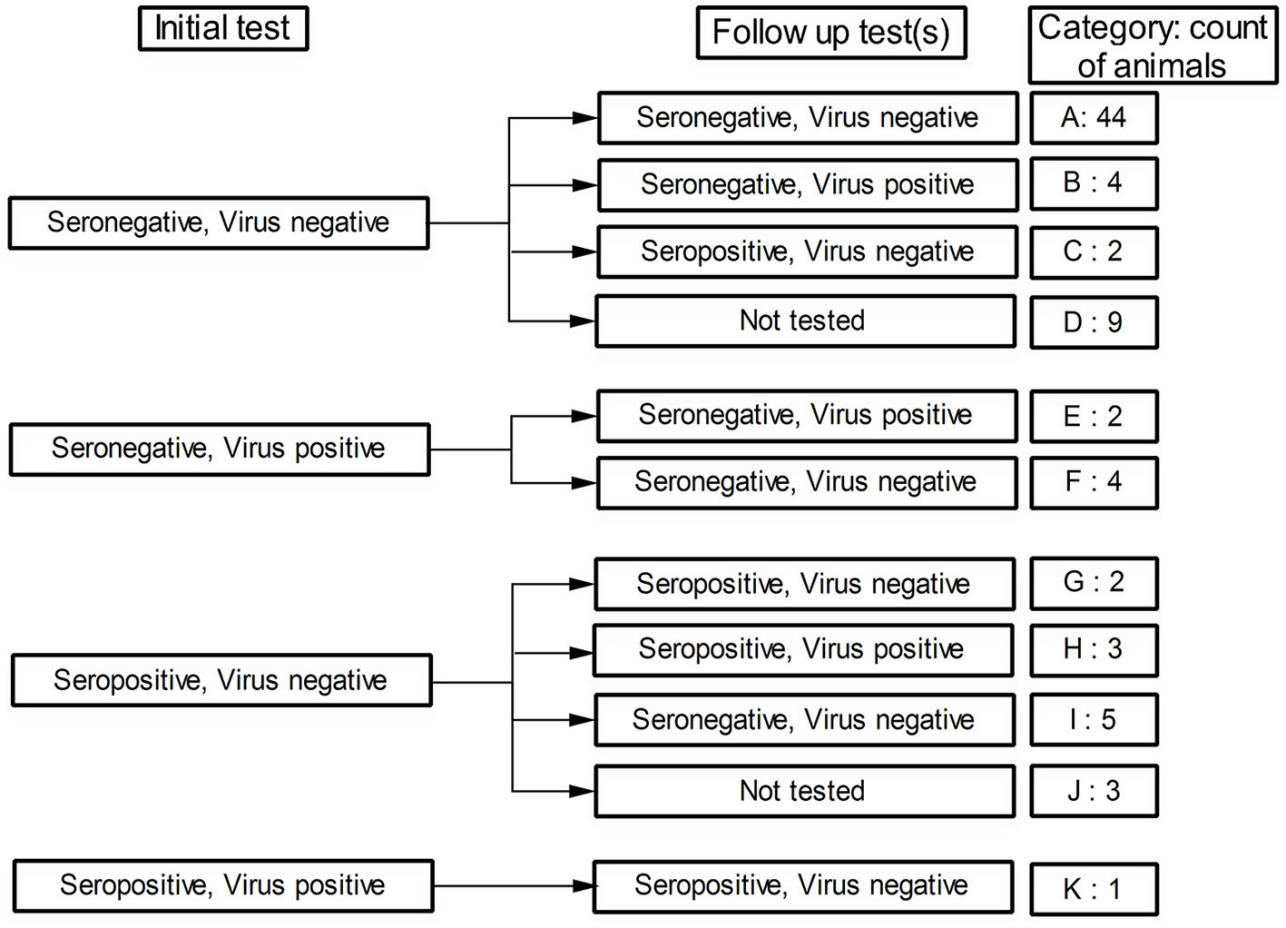
Status of the followed animals regarding BVDV seropositivity and virus detectability (by real-time RT-PCR) in their initial test and follow-up test(s). The category code and number of animals in each category are shown in the last column.

The findings obtained from this follow-up study of the involved animals showed a wide spectrum of scenarios that may be encountered in the BVDV-infected herd. Initial testing of the 79 studied animals showed that 65 animals (82.28%) were seronegative (Figure 2, Categories A to F), some of which (*n* = 6) showed evidence of ongoing BVDV infection as revealed by the results of the real-time RT-PCR (Categories E and F). On the other hand, 14 animals (17.72%) were seropositive at their initial testing (Categories G to K), one of which was also positive in the real-time RT-PCR test (Category K).

Follow-up testing of the initially seronegative/BVDV-negative animals showed that 44 animals maintained their negative status (Figure 2, Category A), 4 other animals remained seronegative but the BVDV nucleic acids were detected in their sera (Category B), 2 additional animals were seroconverted though BVDV was not detected in their sera (Category C), and 9 animals were not followed up due to their early removal or recent involvement in the study (Category D). Regarding the primarily seronegative/BVDV-positive animals, none of these animals were seroconverted (Categories E and F). Moreover, two of these animals maintained their positivity toward the BVDV (Category E).

The follow-up testing of the initially seropositive/BVDV-negative animals revealed that five of these animals either maintained their initial status (Figure 2, Category G) or turned seropositive/BVDV-positive (Category H). The other five animals, most of whom (four out of five) were newborns at the initial test, were turned seronegative/BVDV-negative (Category I). The remaining three animals were not followed up (Category J) due to either death or recent involvement in the study. Finally, the single seropositive/BVDV-positive animal (Category K) sustained its seropositive status and turned BVDV-negative.

Regarding the BVDV incidence rate, two animals were initially seronegative but turned seropositive in the follow-up tests (Figure 2, Category C). The total time of the seronegative animals (animals at risk) was 29,868 cow days. The incidence rate was estimated at 0.00006696 (CI95%: 0.00000811 to 0.00024189) new cases per day, 0.002038 (CI95%: 0.000247 to 0.007362) new cases per month, or 0.02446 (CI95%: 0.00296 to 0.08835) new cases per year.

**Figure 3:**
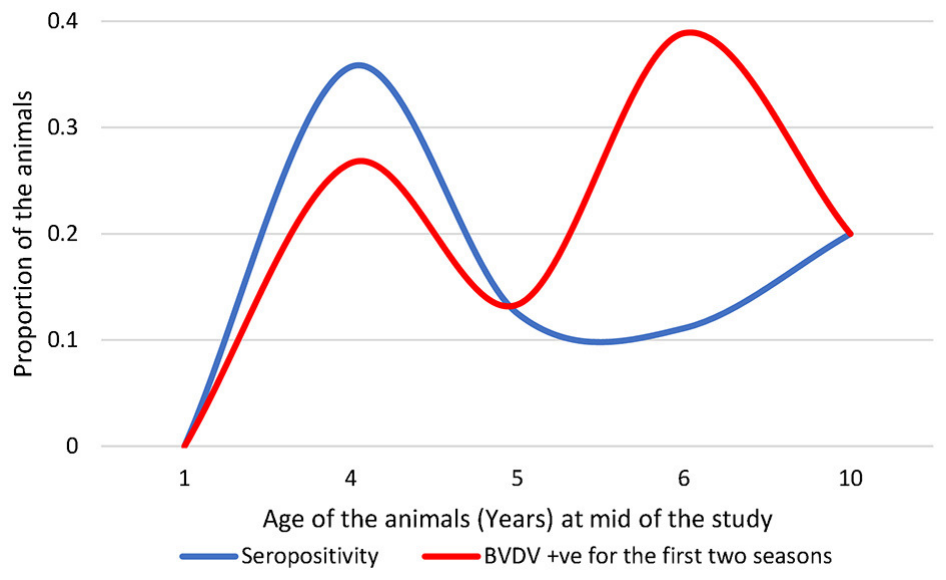
Distribution of the seropositive and BVDV-positive animals over the ages of the studied animals; note that seropositive newborns were not included.

The history of exposure to BVDV infection may be recognized by plotting the seropositivity and BVDV detectability against the ages of the involved animals (Figure 3). BVDV was largely detected in animals aged ~6 years. The BVDV seropositivity was mainly concentrated approximately the age of 4 years, with relatively high BVDV detectability. None of the newborns enrolled during the study showed seropositivity or BVDV, with the exception of the proposed maternal immunity-derived seropositivity (see below). Consequently, seropositivity in newborns was not included in the subsequent analysis.

**Figure 4:**
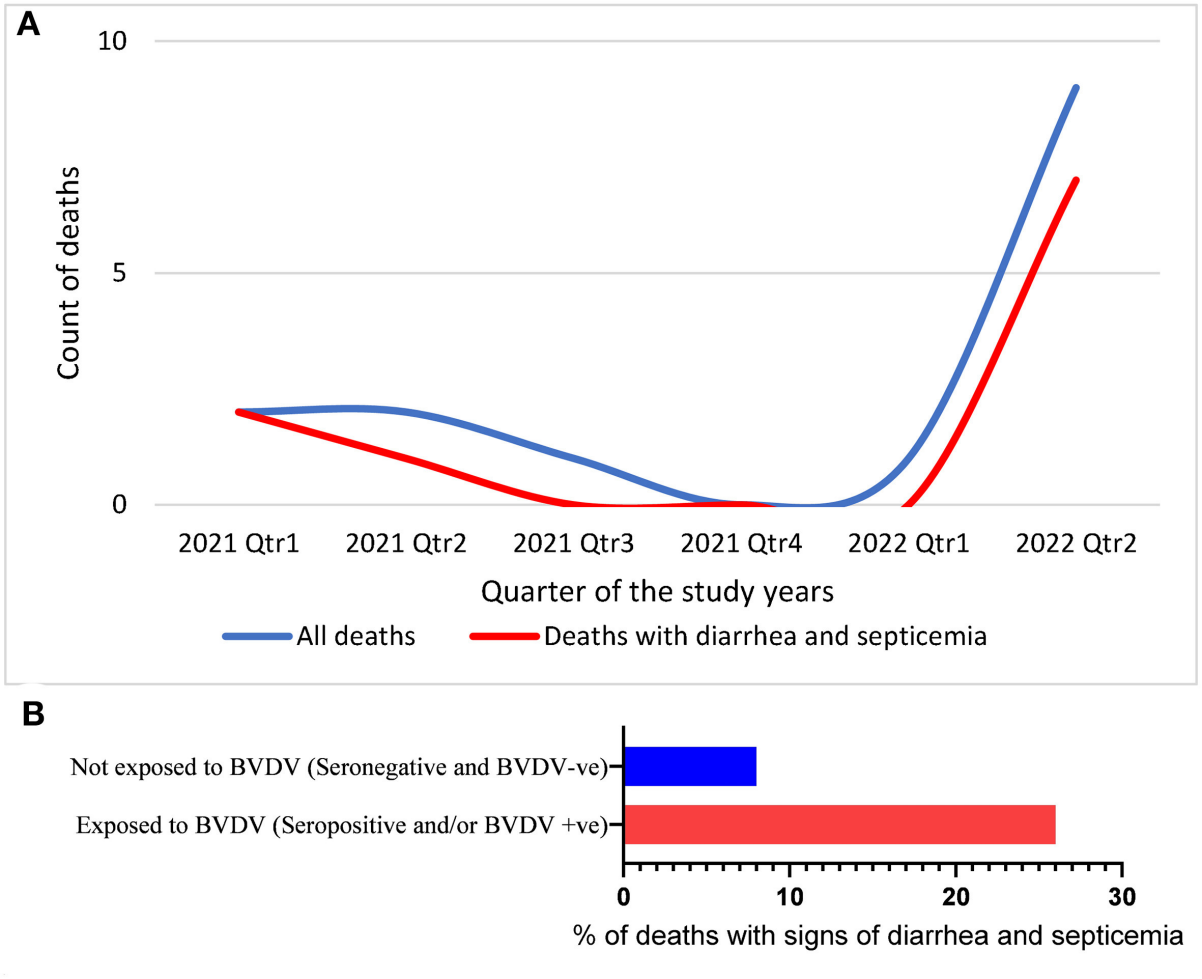
Temporal distribution of deaths **(A)** and the percentage of the animals that died with signs of diarrhea and septicemia out of the animals exposed to BVDV or not exposed to BVDV **(B)**.

During this study, 15 out of 79 animals died, 10 of which showed signs of diarrhea and septicemia. The temporal distribution of the deaths showed considerable elevation in the last 3 months of the study, as shown in Figure 4A. Trying to connect these mortalities with BVDV infection showed that BVDV was detected in three of these deaths, while three were seropositive (including one seropositive and BVDV positive). There was no statistical difference in the distribution of mortalities over categories of serological status (*P*-value = 0.1094). However, the odds of being seropositive were 3.79 times higher among dead animals than alive animals (Table 3). Similarly, there was no statistical connection between mortalities and BVDV detectability (*P*-value 0.3505). The odds of being BVDV-positive were 2.6 times higher among dead animals than living animals. Nevertheless, there was a significant association between mortality and exposure to BVDV (both seropositive and BVDV positives) (*P*-values of chi-square = 0.0399; Mantel–Haenszel = 0.041212; Mid-P exact test = 0.0325; Fisher's exact test = 0.0546). The odds of being exposed to BVDV were 3.93 times higher among dead animals than alive animals (Figure 4B and Table 1).

**Table 1:** Association of mortalities with BVDV seropositivity, BVDV detectability, or both.

	**Died**	**Alive**	**Total**	**Test**	**Value**	**95% CI**
**Mortality vs. seropositivity (seropositivity in newborn was not included)**						
Seropositive	3 (30%)	7 (70%)	10	Fisher's exact test *P*-value	0.1094	
Seronegative	7 (10%)	62 (90%)	69	Odds ratio	3.796	0.8864 to 16.51
Total	10 (13%)	69 (87%)	79	Relative Risk	2.957	0.8932 to 8.398
**Mortality vs. BVDV detections**						
BVDV +ve	3 (25%)	9 (75%)	12	Fisher's exact test *P*-value	0.3505	
BVDV -ve	7 (11%)	55 (89%)	62	Odds ratio	2.619	0.6335 to 10.29
Total	10 (14%)	64 (86%)	74	Relative Risk	2.214	0.6648 to 6.480
**Mortality vs. BVD Exposure (seropositivity and BVDV detection)**						
Exposed	5 (26%)	14 (74%)	19	Fisher's exact test *P*-value	0.0546	
Not exposed	5 (8%)	55 (92%)	60	X^2^, df, *P*-value	4.221, 1, 0.0399	
Total	10 (13%)	69 (87%)	79	Odds ratio	3.929	1.035 to 14.70
				Relative Risk	3.158	1.050 to 9.114

Note that the proposed maternally derived seropositivity was not included.

### 3.5. Association between BVDV exposure and age, gender, and reproductive performance

**Figure 5:**
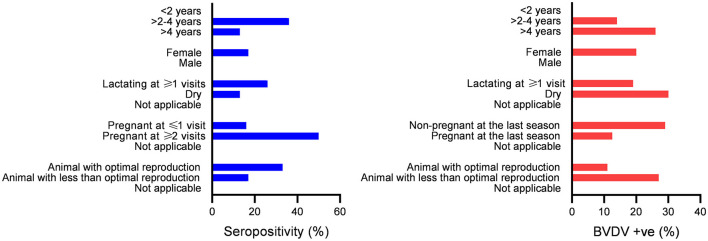
Association between seropositivity or BVDV positivity with categories of the studied factors; bars indicate the number of animals in the categories shown on the Y axis. Significant differences in the distribution of seropositivity or BVDV positivity over categories (*P*-values ≤ 0.05) were found for all studied factors except for gender.

Generally, the present data showed a linkage between seropositivity and reproduction (pregnancy and lactation). In contrast, there was a linkage between BVDV detection and poor reproduction (Figure 5, Tables 2, 3).

**Table 2:** Statistical analysis of the distribution of seropositives across categories of studied factors (age, gender, lactation, and pregnancy).

**Category**	**2020**		**2021**		**2022**		**Overall**				**Statistics for the overall result**	
	+ve^*^	**-ve** ^**^	+**ve**	**-ve**	+**ve**	**-ve**	**Category**	+**ve**	**-ve**	**Total**	**Test**	***P*** **-value**
**Age (at the end of the year 2021)**												
< 2 years	5	11	1	13	0	22	< 2 years	0 (0%)	26 (100%)	26	Fisher's test	0.003988061
>2–4 years	5	9	4	10	3	7	>2–4 years	5 (36%)	9 (64%)	14	Fisher's test Mid-P	0.003588019
>4 years	3	36	4	28	2	27	>4 years	5 (13%)	34 (87%)	39	Chi-square	0.0052
Total	13	56	9	51	5	56	Total	10 (13%)	69 (87%)	79		
**Gender**												
Female	11	46	8	46	5	38	Female	10 (17%)	50 (83%)	60	Fisher's test	0.10689024
Male	2	10	1	5	0	18	Male	0 (0%)	19 (100%)	19	Fisher's test Mid-P	0.080724125
Total	13	56	9	51	5	56	Total	10 (13%)	69 (87%)	79	Chi-square	0.0569
**Lactation**												
Dry	4	27	7	26	4	21	Lactating at ≥1 season of study	7 (26%)	20 (74%)	27	Fisher's test	0.007147168
Lactating	4	15	1	12	1	10	Dry	3 (13%)	20 (87%)	23	Fisher's test Mid-P	0.006601349
Not applicable	5	14	1	13	0	25	Not applicable	0 (0%)	29 (100%)	29	Chi-square	0.0142
Total	13	56	9	51	5	56	Total	10 (13%)	69 (87%)	79		
**Pregnancy**												
Not pregnant	6	38	6	32	3	18	Pregnant at ≤ 1 season	7 (16%)	37 (84%)	44	Fisher's test	0.002511834
Pregnant	2	4	2	6	2	13	Pregnant at ≥2 seasons	3 (50%)	3 (50%)	6	Fisher's test Mid-P	0.002245845
Not applicable	5	14	1	13	0	25	Not applicable	0 (0%)	29 (100%)	29	Chi-square	0.0341
Total	13	56	9	51	5	56	Total	10 (13%)	69 (87%)	79		
							Overall reproductive performance (Lactation and pregnancy)					
							Animals with optimal reproduction	3 (33%)	6 (67%)	9	Fisher's test	0.006857615
							Animals with less than optimal reproduction	7 (17%)	34 (83%)	41	Fisher's test Mid-P	0.006202202
							Not applicable	0 (0%)	29 (100%)	29	Chi-square	0.015
							Total	10 (13%)	69 (87%)	79		

Overall calculation considered the results of the three sampling seasons for individual animals. Note that the proposed maternally derived seropositivity is shown in the year column but not included in the analysis of the overall results.

^*^+ve, seropositive.

^**^-ve, seronegative.

**Table 3:** Statistical analysis for the distribution of BVDV positivity in real-time RT-PCR and studied factors (age, gender, lactation, and pregnancy).

**Category**	**2020**		**2021**		**Overall**				**Statistics for the overall result**	
	+ve^*^	**-ve** ^**^	**ve** ^*^	**-ve** ^**^	**Category**	**ve** ^*^	**-ve** ^**^	**Total**	**Test**	***P*** **-value**
**Age (at the end of the year 2021)**										
< 2 years	0	16	0	14	< 2 years	0 (0%)	21 (100%)	21	Fisher's test	0.022768157
>2–4 years	1	13	1	13	>2–4 years	2 (14%)	12 (86%)	14	Fisher's test Mid-P	0.021449968
>4 years	6	33	5	27	>4 years	10 (26%)	29 (74%)	39	Chi-square	0.0359
Total	7	62	6	54	Total	12 (16%)	62 (84%)	74		
**Gender**										
Female	7	50	6	48	Female	12 (20%)	48 (80%)	60	Fisher's test	0.106778996
Male	0	12	0	6	Male	0 (0%)	14 (100%)	14	Fisher's test Mid-P	0.074894322
Total	7	62	6	54	Total	12 (16%)	62 (84%)	74	Chi-square	0.0675
**Lactation**										
Dry	6	25	3	30	Lactating at ≥1 season of the study	5 (19%)	22 (81%)	27	Fisher's test	0.008320206
Lactating	1	18	3	10	Dry	7 (30%)	16 (70%)	23	Fisher's test Mid-P	0.007869252
Not applicable	0	19	0	14	Not applicable	0 (0%)	24 (100%)	24	Chi-square	0.0168
Total	7	62	6	54	Total	12 (16%)	62 (84%)	74		
**Pregnancy**										
Not pregnant	5	39	6	32	Non-pregnant at the last season^***^	11 (29%)	27 (71%)	38	Fisher's test	0.003171351
Pregnant	2	4	0	8	Pregnant at the last season	1 (12.5%)	7 (87.5%)	8	Fisher's test Mid-P	0.002952007
Not applicable	0	19	0	14	Not applicable	0 (0%)	28 (100%)	28	Chi-square	0.0321
Total	7	62	6	54	Total	12 (16%)	62 (84%)	74		
					Overall reproduction (Lactation and pregnancy)					
					Animals with optimal reproduction	1 (11%)	8 (89%)	9	Fisher's test	0.010725571
					Animals with less than optimal reproduction	11 (27%)	30 (73%)	41	Fisher's test Mid-P	0.01007767
					Not applicable	0 (0%)	24 (100%)	24	Chi-square	0.0164
					Total	12 (16%)	62 (84%)	74		

Overall calculation considered the results of the first and second sampling seasons for individual animals.

^*^+ve, BVDV positive.

^**^-ve, BVDV negative.

^***^The sample collected at the end of the year 2021 was the last completely tested sample set.

#### 3.5.1. Association of BVDV exposure with the age of the studied animals

There was a significant difference in the distribution of seropositive over age strata (*P*-value = 0.004), with higher seropositivity (36%) in the >2-to-4-year age strata compared to 13% in the age strata >4 years. Additionally, there was a significant difference in the distribution of BVDV positives over age strata (*P*-value = 0.023), with higher BVDV positivity in age strata of >4 years (26%) compared to 14% in the age strata of >2 to 4 years. Regarding BVDV exposure in the strata < 2 years, all animals in these strata were added as newborns. In the year 2020, 16 newborn animals were added, out of which 5 were seropositive and turned seronegative in the following year (*n* = 4) or were not tested (*n* = 1). None of the animals in this group were seroconverted or showed evidence of BVDV infection by real-time RT-PCR. In the year 2021, five newborns were added, one of which was seropositive and not followed (sold), while the others were seronegative. None of these animals were BVDV-positive or seroconverted. In the year 2022, five newborns were added, none of which were seropositive or BVDV-positive.

#### 3.5.2. The correlation between BVDV exposure and the gender of the studied animals

There was no significant difference in the distribution of seropositivity (*P*-value = 1069) or BVDV positives (*P*-value = 1068) over the gender categories of the studied animals.

#### 3.5.3. The correlation between BVDV exposure and lactation status of the studied animals

There was a significant difference in the distribution of seropositivity over lactation categories (*P*-value = 0.0071), where those lactating for one season of the study or more showed higher seropositivity (26%) compared to dry animals (13%) or animals without relevance to lactation (0%). Similarly, there was a significant difference in the distribution of BVDV positivity over lactation categories (*P*-value = 0.0083), where dry animals showed higher BVDV positivity (30%) compared to those lactating for one season or more (19%) or those not relevant to lactation (0%) as shown in Figure 5 and Tables 2, 3.

#### 3.5.4. The correlation between pregnancy and exposure to BVDV infection in the current study

There was a significant difference in the distribution of seropositivity over pregnancy categories (*P*-value = 0.0025), where those pregnant for two seasons of the study or more showed higher seropositivity (50%) compared to those pregnant for one season or less (16%) or those not related to pregnancy (0%). Similarly, there was a significant difference in the distribution of BVDV positivity over pregnancy categories (*P*-value = 0.0032), where those not pregnant at the time of collection of the 2021 sample (the last sample set completely tested for BVDV by real-time RT-PCR) showed higher BVDV positivity (29%) compared to those pregnant (12.5%) or those without relevance to pregnancy (0%).

#### 3.5.5. The correlation between BVDV exposure and the reproduction status of the studied animals

There was a significant difference in the distribution of seropositivity over reproduction categories (*P*-value = 0.0069), where those with optimal reproduction (either pregnant or lactating at the three seasons) showed higher seropositivity (33%), compared to those with lower reproductive performance (17%) or those not related to pregnancy and lactation (0%). Similarly, there was a significant difference in the distribution of BVDV positivity over reproduction categories (*P*-value = 0.0107), where those with lower reproductive performance (dry and non-pregnant on at least one season) showed higher BVDV positivity (27%), compared to those with optimal reproduction (11%), or those not appropriate for pregnancy and lactation (0%).

## 4. Discussion

BVDV infection has significant economic impacts on the dairy and feedlot cattle industries worldwide, including in the Gulf area. The development of a BVDV control/prevention strategy requires prior knowledge of certain epidemiological parameters, such as the incidence rate and the risk factors affecting BVDV transmission. This report presents the findings of a long-term follow-up study to determine the incidence of BVD and some of the associated factors in a dairy herd in the Eastern Region of Saudi Arabia.

In the present study, seroprevalence ranged between 18.8% and 8.2% in the first and last years of the study, respectively. Previous reports showed that the seroprevalence of BVD varies depending on the geographic region and the management practices of the cattle (34). The prevalence of BVD can range from < 2% to 97% in cattle herds (35). A seroprevalence of 26% and 35% was previously reported in Saudi Arabia (27, 28). Similarly, seroprevalence ranging from 25% to 80% was reported from Jordan, Sudan, Iraq, Oman, Egypt, Iran, India, and Bangladesh (22, 24–26, 29, 36–38).

In the present study, BVDV was detected in serum samples from 10 seronegative adult animals on single (*n* = 6) or dual occasions (*n* = 4) (categories B, E, and F). Additionally, two of these animals died with signs of diarrhea and septicemia at the age of 6 years (category F). By definition, a BVDV-PI animal is one that has tested positive on two consecutive occasions, 3 weeks or more apart (11, 12). It is well-documented that BVDV-PI animals are immunotolerant and seronegative for the causative BVDV strain (6). On the contrary, after BVDV infection in immunocompetent animals, seroconversion that is maintained for at least 3 years is expected (39). Thus, viremic animals in the first/second years of the current study were expected to become seropositive in the following year. None of these animals were seroconverted and, hence, were possibly BVDV-immunotolerant. Collectively, at least the four seronegative animals with dual virus detections in the present study were probably PI animals. The cons for this scenario are the age of the possibly PI animals and the low intra-herd transmission pattern observed.

All 10 seronegative BVDV-positive animals were adults (ages 4–10 years). Previous studies showed that the possibility of detecting PI animals decreases with increasing age (10). Previous studies also reported high mortality rates in PI animals, where these animals may develop mucosal disease (MD) while being young (1 to 2 years). Typically, outbreaks of MD start with the death of a PI animal, usually at the age of 6 to 24 months, and subsequent testing of the affected group reveals some seropositive and some seronegative-BVDV-positive animals (40). However, some of the PI animals may remain alive and may give birth to PI calves (41, 42). Previous studies detected PI at the age of 4 years (43) and showed the failure to induce MD by the administration of adrenocorticotropic hormone into PI animals at the age of 36 months (44). This may give an impression of how long a PI animal might survive. In the present study, 10 animals died, ranging in age from a month to 6 years, with signs of diarrhea and septicemia. Such deaths in animals that were exposed to BVDV (either seropositive or BVDV-positive) were significantly higher than those in animals that were not exposed (seronegative and BVDV-negative). However, the etiology of the diarrhea and septicemia in these animals was not identified, a limitation of the current study.

In the present study, low intra-herd transmission was observed as only two animals were seroconverted during the first season (Category C). Meanwhile, young animals and bulls remained apparently uninfected. No animals were seroconverted during the last year of the study. Seroprevalence ranged from 18.8% in the first year to 8.2% in the last year of the study. Additionally, none of the tested nasal and rectal swabs were positive for BVDV antigen or nucleic acid, suggesting minimal virus shedding. The incidence rate was estimated at 0.00006696 new cases per day. Intra-herd transmission patterns like those found in this study were previously reported to occur in the presence of TI animals rather than PI animals. Typically, the pattern of distribution of seropositive animals over animal age showed that all animals born after the removal of the PI animals remained seronegative (45). In the presence of TI animals, transmission was limited and ceased within 30 months. On the other hand, in the presence of PI calves, 90% or higher of surrounding susceptible animals became infected within 3 to 6 months (19, 42, 46). Similarly, (47) reported an average seroprevalence of 87% in herds with one or more PI animals, while seroprevalence was averaged at 43% in herds without PI animals. In an intensive management system with an animal density of 67 animals/km^2^, the basic reproductive number (*R*_0_) was estimated at *R*_0_ = 35 if 1.2% of the animals were PI animals and at *R*_0_ = 2.3 in the absence of the PI animals, suggesting an ~15-fold increase in transmission rate in the presence of PI animals (48). Regarding the incidence rate, it has been reported that under a grazing situation with a density of 0.2 to 1 animal/acre, the incidence rate in the presence of TI animals was not observed, while it was 0.006 to 0.04 new cases per day in the presence of PI animals and increased to 1.2 new cases per day if PI animals were housed with susceptible animals (49). Hence, besides the presence of PI animals, the separation distance and animal density are other key factors affecting the incidence rate (19).

As just mentioned, the role of the TI animals in the transmission of BVDV remained ambiguous, as revealed by the reported inconsistent findings that ranged from no incidence to the maintenance of the infection for a few years (46, 49). This is supported by the experimental findings showing that BVDV was readily transmissible from PI animals to susceptible animals after close contact, while transmission from TI animals to susceptible animals was not observed after close contact for up to day 42 post-infection (50). Subsequently, either extended intermittent viral shedding by the TI animals (>2 weeks) or intermittent viral shedding by the PI animals was suggested to resolve this puzzle (51). Succeeding studies have clarified some of these aspects. In those studies, BVDV was detected by means of real-time RT-PCR in blood components at >30 days (52), 85 days (53), and 98 days (54) after acquiring the TI. Blood transfusions from these animals into susceptible animals resulted in the transmission of the BVDV infection (54). Consequently, the dual-viremic animals described in the present study were possibly TI animals with extended viral persistence. This is also in agreement with previous reports showing that BVDV may maintain circulation for long periods with no PI animals (55). The cons of this scenario are the long interval (6–11 months) separating the dual virus detections and the seronegativity of these animals.

Alternatively, the presence of viremia at multiple time points may be attributed to reinfection with BVDV. In support of this scenario is the introduction of new animals into the studied herd and the reinfection of the previously seropositive, BVDV-negative animals (Category H). Additionally, the seronegativity of dual-viremic animals suggests that they were not able to develop immunity and thus remained susceptible to reinfection with BVDV. Previous studies showed the existence of high genetic diversity in BVDV (56). In these regards, simultaneous natural infection with BVDV-1 and BVDV-2 in cattle was previously reported (57). Furthermore, simultaneous natural infection with subtypes 1a and 1b was detected in buffalo (58), and co-circulation of different genotypes and subtypes of BVDV in a single cattle herd was also reported (59). As stated earlier, BVDV infection usually induces a protective immune response that lasts for years. However, cross-protection between strains of different BVDV types was reported to be weak, while it was variable between strains of the same BVDV type (60, 61). Peripheral mononuclear cells (PMNCs) from BVDV-immune animals were susceptible to *in vitro* infection with homologous and heterologous strains (62). BVDV can also persist in peripheral blood leukocytes from seropositive animals, despite the ability of the sera of these animals to neutralize the isolated virus (54, 63).

Several studies reported a higher sensitivity of the RT-PCR test than the AC-ELISA for the identification of BVDV-infected animals (64). In the present study, the lack of BVDV antigen in the serum of the PCR-positive animals may indicate the circulation of a distinct virus strain that is not covered by the used AC-ELISA kit. This interpretation is supported by previous studies reporting different capacities of commercial ELISA kits to detect BVDV antigen from different virus strains. One could speculate that, as this assay usually depends on monoclonal antibodies, a minor antigenic change may lead to a false-negative, as previously reported (65).

Considering the age strata of the studied animals, BVDV was not detected in the newly born animals, while five of these animals were seropositive at the age of 1–6 months (Categories B and I). Seropositivity in the newly born animals could be attributed to maternal immunity, as all of these calves were seronegative in the following year except one animal that was not tested. This is in agreement with previous studies showing that anti-BVDV maternal antibodies disappear within the first 6 months of life (42, 46). Similarly, it has been shown that maternal immunity-derived protection may persist for up to 9 months (4).

Variable factors were connected to BVD, such as age, sex, pregnancy, and lactation. In the present study, seropositivity was higher in adults than in young animals, in lactating than in dry animals, and in pregnant than in non-pregnant animals. An opposite association was found with BVDV positivity. A similar association pattern across all factors was previously reported (66). Concerning the association with age, similar findings were also reported by others (47, 67, 68). Regarding gender, our findings agree with those reported by (68), where no difference was found between males and females.

## 5. Conclusion

To the best of our knowledge, this is the first longitudinal study of BVDV among some cattle populations in the Eastern Region of Saudi Arabia. Viremia was detected once or twice with a long interval in some animals that remained seronegative, suggesting the possibility of being PI animals. The observed low incidence rate and the age of these animals (4 to 10 years) indicate that these animals were rather TI animals. The reported findings shed light on the possibility of prolonged persistence of BVDV in TI animals and/or the possibility of BVDV reinfection. Additionally, it showed the high frequency of seronegativity in exposed animals. Further studies with stronger biosecurity and shorter sampling intervals are needed to uncover the nature of BVDV transmission and allow unambiguous discrimination between PI and TI BVDV infections. Large-scale studies are also encouraged to study in detail the molecular epidemiology and pathogenesis of BVDV in the Gulf region.

## Data availability statement

The original contributions presented in the study are included in the article/supplementary material, further inquiries can be directed to the corresponding authors.

## Ethics statement

The animal studies were approved by King Faisal Animal Ethics Committee. The studies were conducted in accordance with the local legislation and institutional requirements. Written informed consent was obtained from the owners for the participation of their animals in this study.

## Author contributions

Conceptualization, software and validation, data curation, visualization, supervision, and writing—review and editing: AA-M, AA-K, JH, MK, and MH. Methodology: AA-M, AA-K, AS, JH, MK, BF, and MH. Formal analysis: AA-M, AA-K, JH, MK, and MH. Resources: AA-M and MH. Writing—original draft preparation: AA-M, AA-K, AS, JH, MK, BF, and MH. Project administration and funding acquisition: AA-M. All authors have read and agreed to the published version of the manuscript.
